# Supplementary material for: Asymmetric genome merging leads to gene expression novelty through nucleo-cytoplasmic disruptions and transcriptomic shock in Chlamydomonas triploids
Source: New Phytol. Author manuscript; Available in PMC 2025 Jul 1. (PMC7616817; doi:10.1111/nph.20249)
Supplement: Supplementary Material [file EMS200016-supplement-Supplementary_Material.docx]

### ***New Phytologist Supporting Information***

###

### **Asymmetric genome merging leads to gene expression novelty through nucleo-cytoplasmic disruptions and transcriptomic shock in *Chlamydomonas* triploids**

Lucas Prost-Boxoen^1,2,3^, Quinten Bafort^1,2,3^, Antoine Van de Vloet^1,2^, Fabricio Almeida-Silva^1,2^, Yunn Thet Paing^1,2^, Griet Casteleyn^1,2,3^, Sofie D’hondt^3^, Olivier De Clerck^3^, Yves Van de Peer^1,2,3,4,5*^

^1^ Department of Plant Biotechnology and Bioinformatics, Ghent University, 9052 Ghent, Belgium

^2^ VIB Center for Plant Systems Biology, VIB, 9052 Ghent, Belgium

^3^ Department of Biology, Ghent University, Ghent, Belgium

^4^ Centre for Microbial Ecology and Genomics, Department of Biochemistry, Genetics

and Microbiology, University of Pretoria, Pretoria 0028, South Africa.

^5^ College of Horticulture, Academy for Advanced Interdisciplinary Studies, Nanjing

Agricultural University, Nanjing, China.

Article acceptance date: 21 October 2024

**
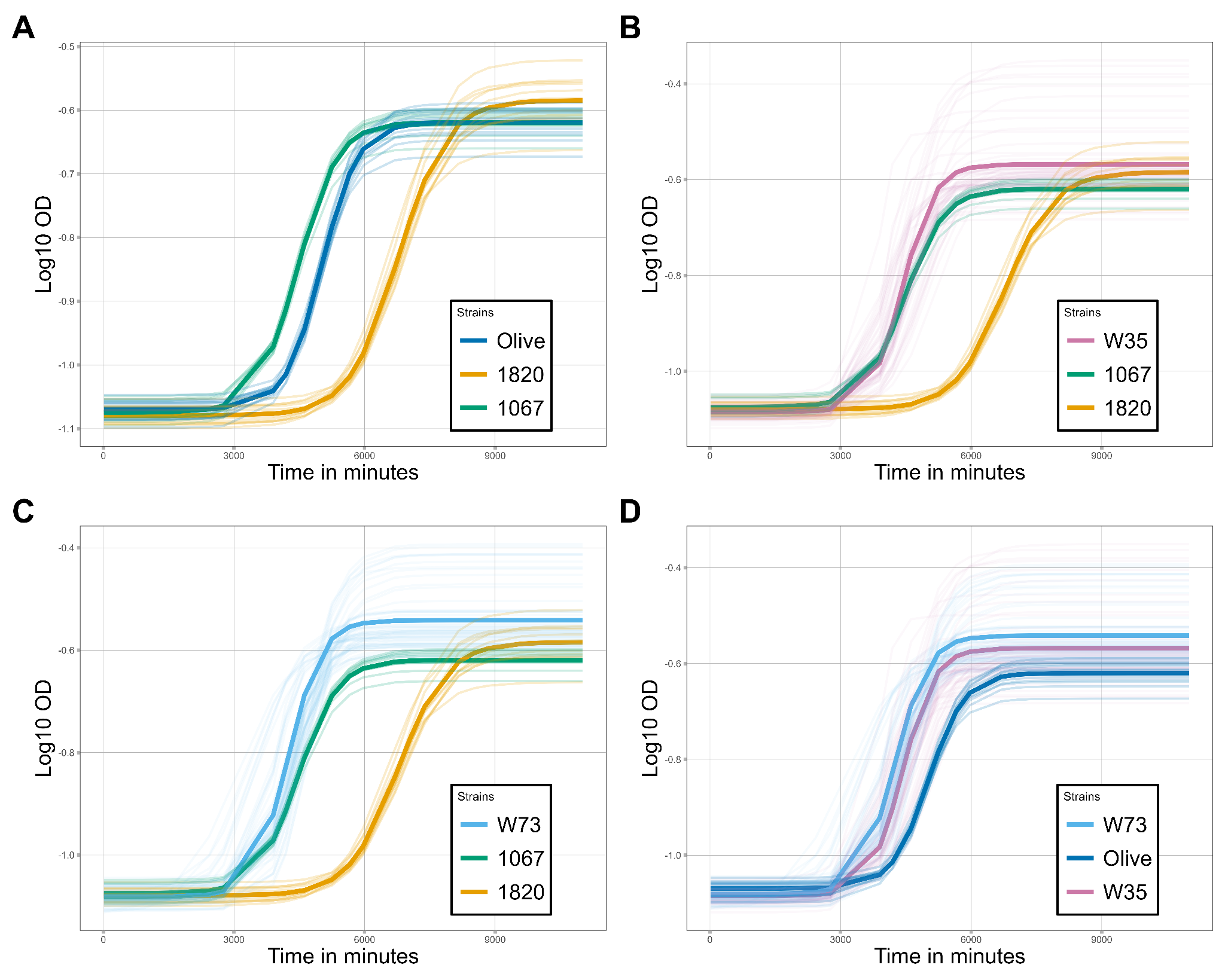
**

**Supplementary Figure S1. Growth curves of the different Chlamydomonas reinhardtii experimental strains.** This plot represent the growth curves of the haploid parent (1N), the diploid parent (2N), the triploid progeny (3N G0) and the triploid lines at generations 225 and 425 (3N G225 and 3N G425 respectively). Transparent lines represent the individual replicates, while bold lines represent the average growth curve for each strain. The growth curves are displayed in four separate plots for clarity and ease of comparison. **A**: growth curves of the two parental strains (1N and 2N) and the triploid progeny (3N G0). **B**: growth curves of the two parental strains (1N and 2N) and the triploid lines at generation 225 (3N G225). **C**: growth curves of the two parental strains (1N and 2N) and the triploid lines at generation 425 (3N G425). **D**: growth curves of the triploid lines at three different time-points, G0, G225 and G425.


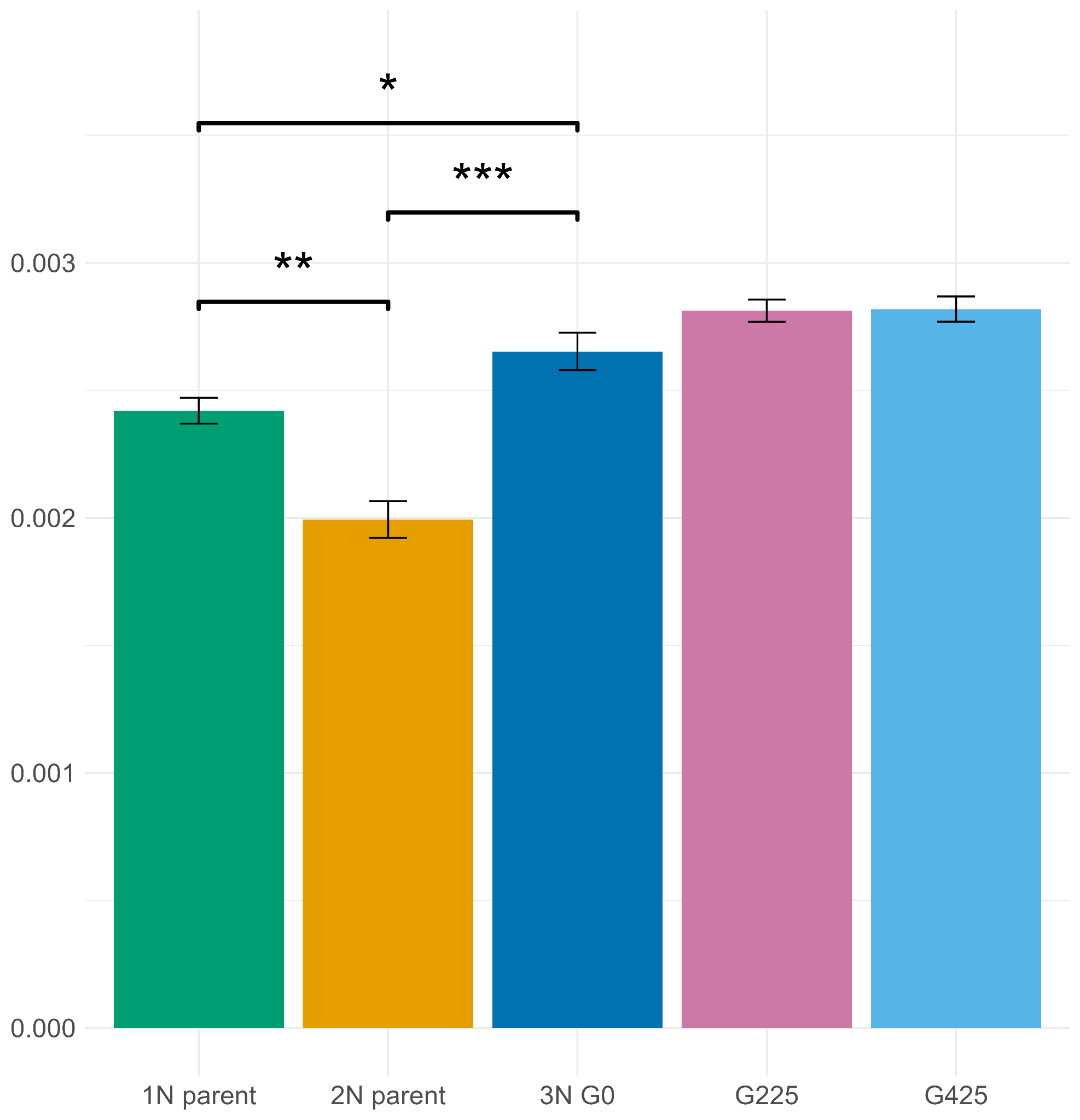


**Supplementary Figure S2.** **Mean maximum growth rate (MGR) of Chlamydomonas reinhardtii parental strains, ancestral triploid and triploid lines pooled at generation time-points (G225 and G425).** Error bars represent standard errors. Presented strains include haploid CC-1067 (1N parent, green), diploid CC-1820 (2N parent, orange), ancestral triploid (3N G0, purple), and a pooled representation of evolved triploid lines (Lines 1-5) at generations 225 (salmon pink) and 425 (blue). Statistical significance of differences between lines, determined by t-tests or Mann-Whitney U tests based on data distribution, is indicated above comparisons (‘ns’ for not significant, ‘*’ for p < 0.05, ‘**’ for p < 0.01, ‘***’ for p < 0.001) (only the 1N parent, the 2N parent and the 3N G0 are compared).


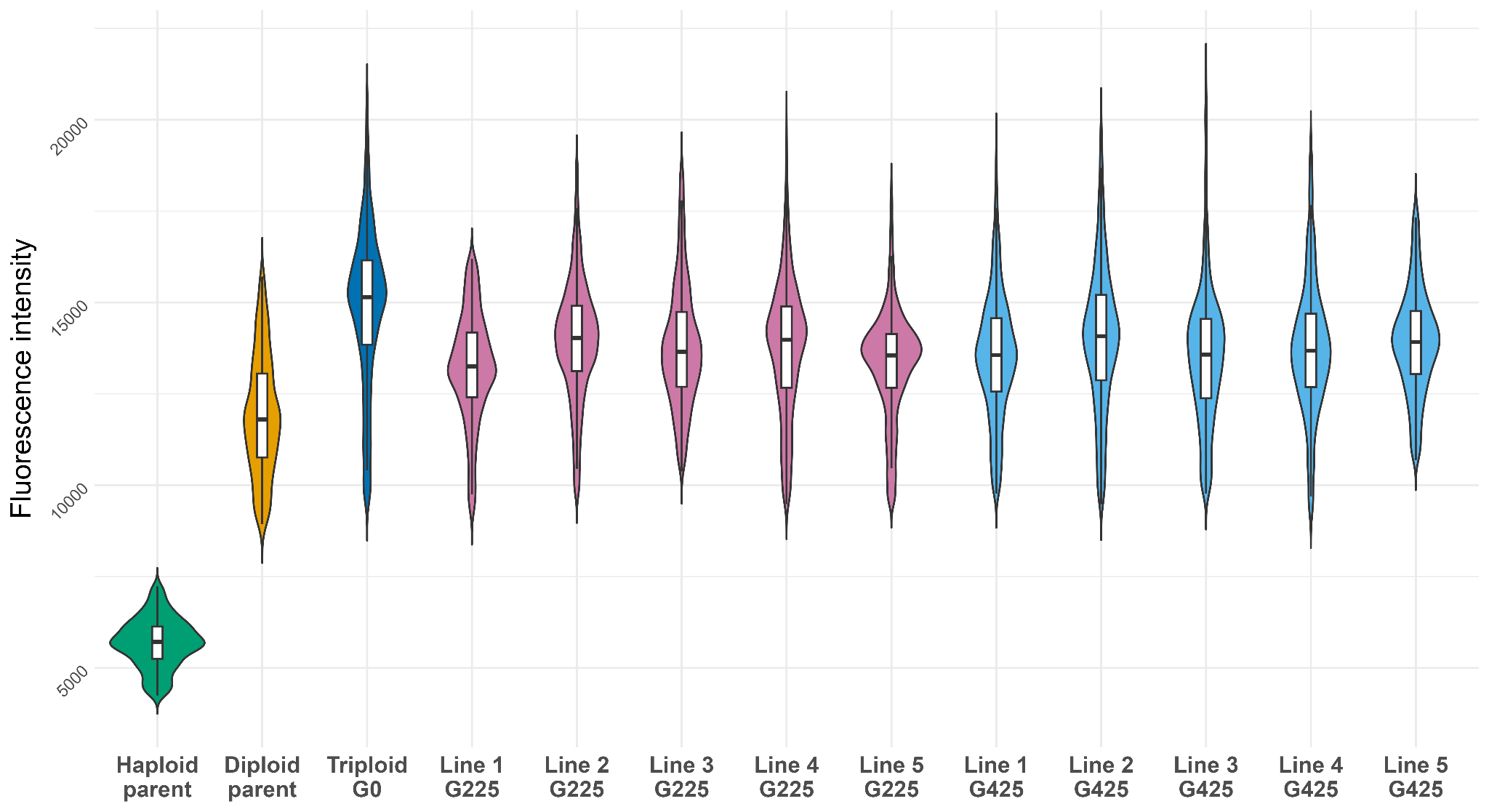


**Supplementary Figure S3. Comparative flow cytometric analysis of propidium iodide (PI)-stained nuclei for genome size estimation in our Chlamydomonas reinhardtii lines.** The violin and box plots display the distribution of fluorescence intensity, indicative of DNA content. Categories include haploid and diploid parent strains, the triploid progeny at generation 0 (G0) and five independently evolving triploid lines at generation 225 and 425 (G225 and G425).


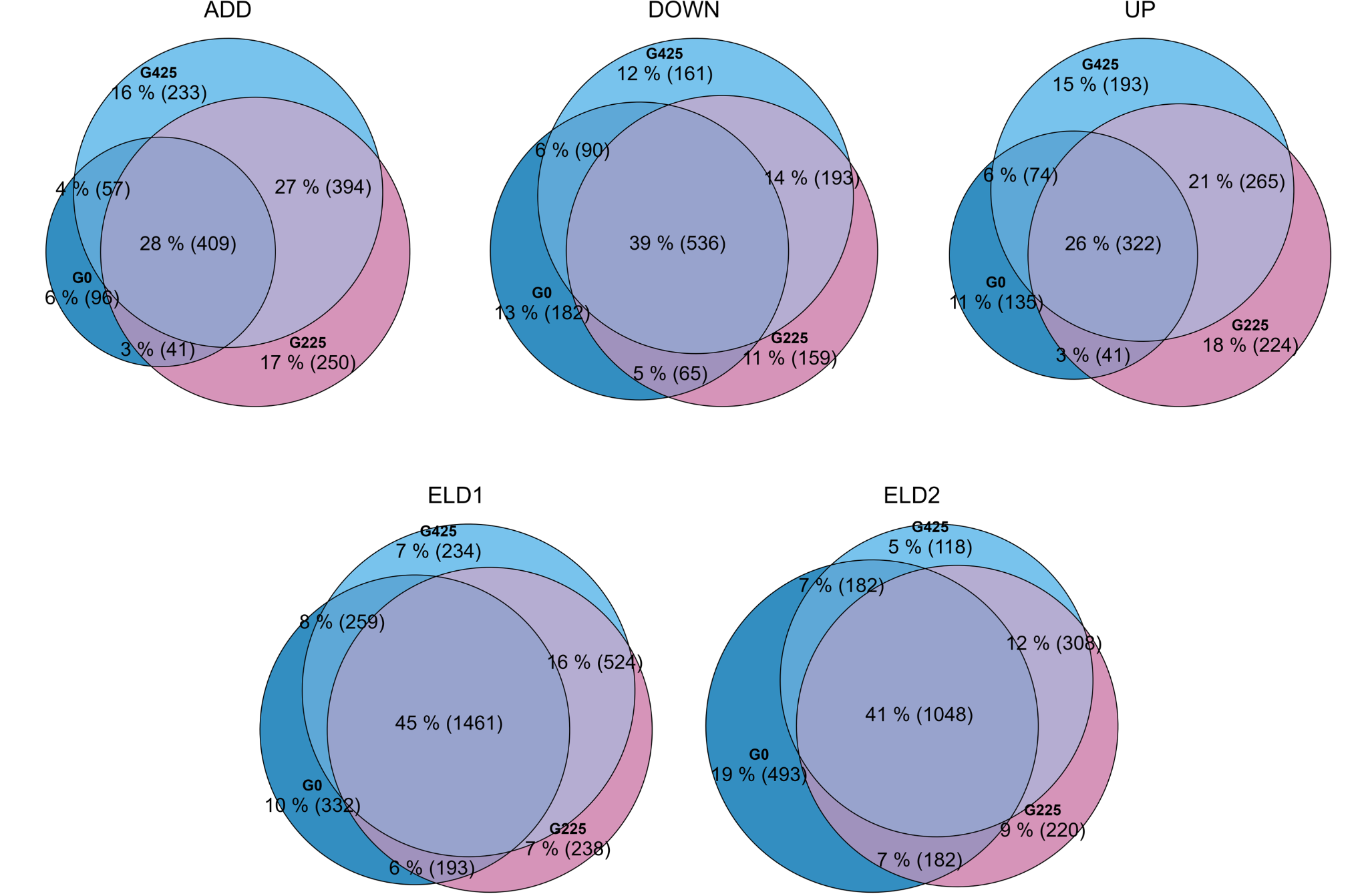


**Supplementary Figure S4. Conservation of expression patterns across three experimental evolution time points (G0, G225, and G425) in the Chlamydomonas reinhardtii triploid progeny lines.** Euler diagrams illustrate the number and percentage of genes consistently represented within the five expression patterns across generation 0 (G0), generation 225 (G225), and generation 425 (G425). The five expression patterns are: transgressive upregulation (UP), transgressive downregulation (DOWN), additivity (ADD), expression level dominance toward the haploid parent (ELD1), and expression level dominance toward the diploid parent (ELD2).


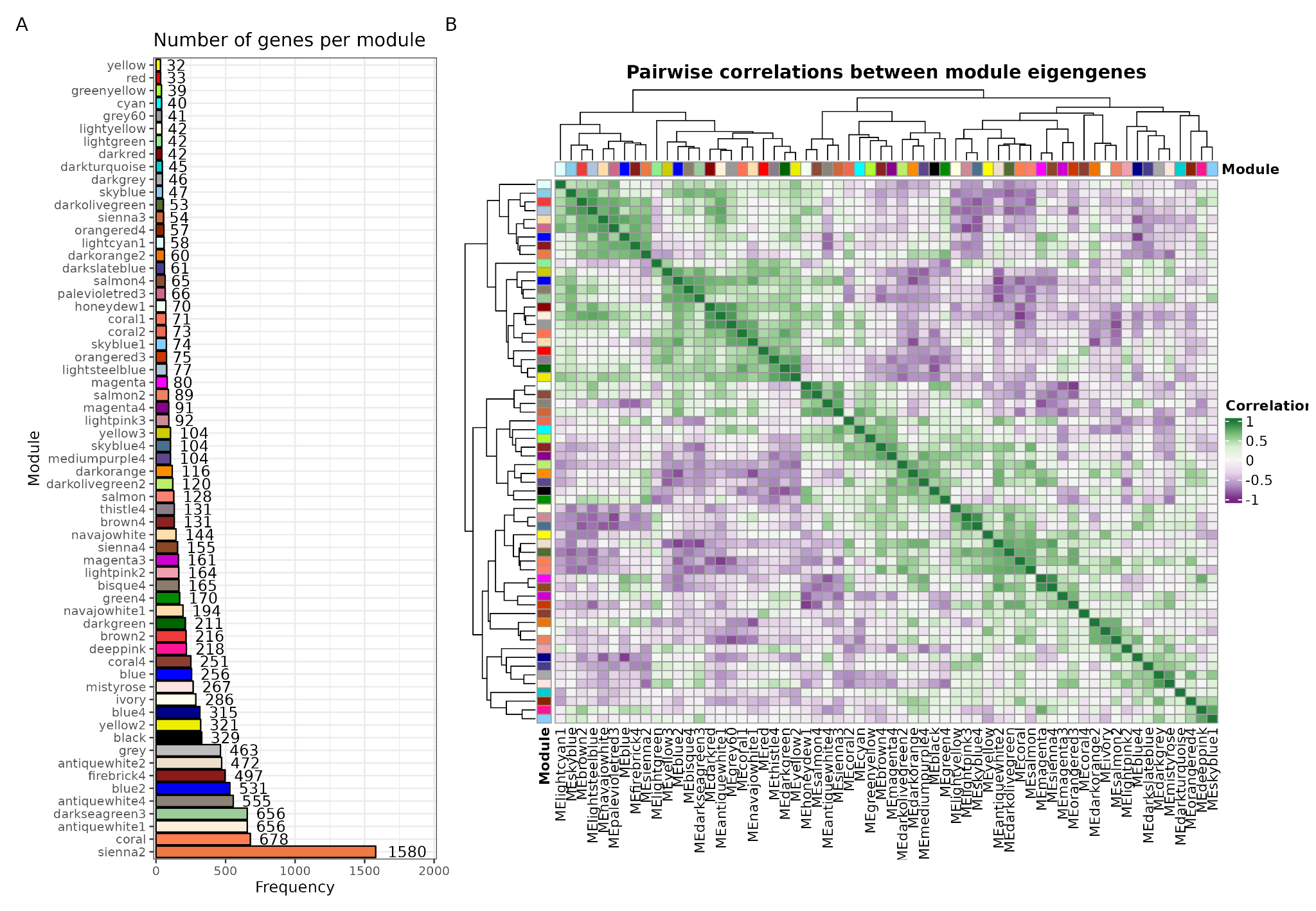


**Supplementary Figure S5.** **Summary statistics of coexpression modules for the network with all** Chlamydomonas reinhardtii **samples.** **A.** Absolute frequency of genes per module. **B.** Pairwise Spearman’s correlations between module eigengenes. Module eigengenes are the first principal component of each module, and they represent a summary of the expression profiles of the entire module.


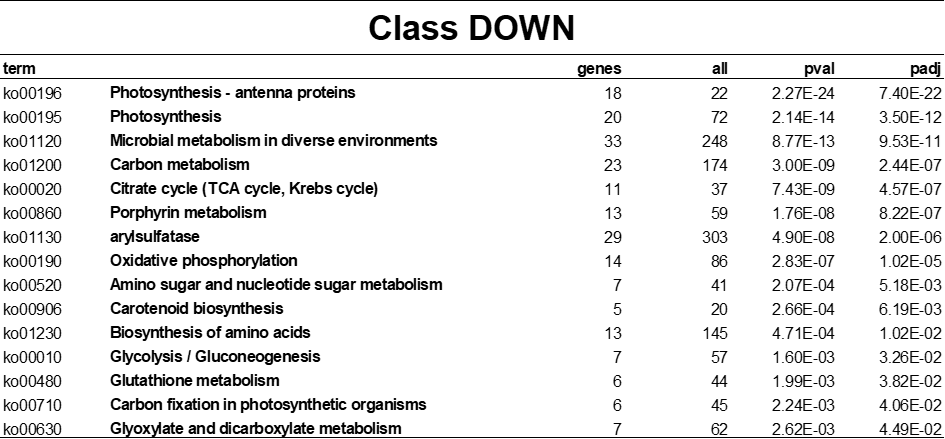

**Supplementary Table 1. Overrepresented KEGG pathways among persistent downregulated genes in the Chlamydomonas reinhardtii triploid.** This table lists KEGG pathways that are significantly overrepresented within genes exhibiting persistent decreased expression levels, organized in ascending order of adjusted p-values to highlight the most statistically significant enrichments.


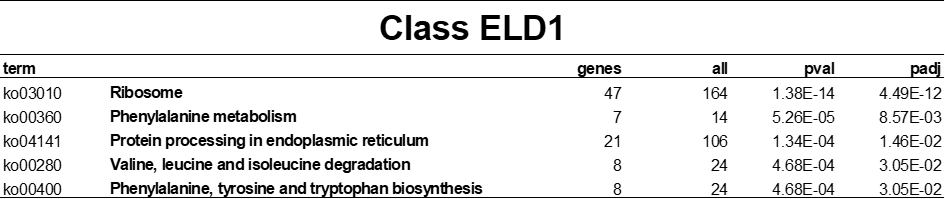


**Supplementary Table 2. Overrepresented KEGG pathways among genes showing persistent expression level dominance towards the Chlamydomonas reinhardtii haploid parent (ELD1).** This table lists KEGG pathways that are significantly overrepresented within persistent ELD1 genes, organized in ascending order of adjusted p-values to highlight the most statistically significant enrichments.


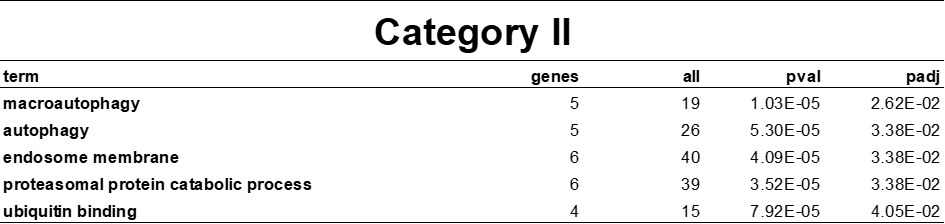


**Supplementary Table 3. Overrepresented GO terms in Category II genes.** This table presents Gene Ontology (GO) terms that are significantly overrepresented among Category II genes–those exhibiting a rapid increase in expression during the laboratory natural selection experiment in the Chlamydomonas reinhardtii triploid lines. The terms are listed in ascending order of adjusted p-values to highlight the most statistically significant enrichments.


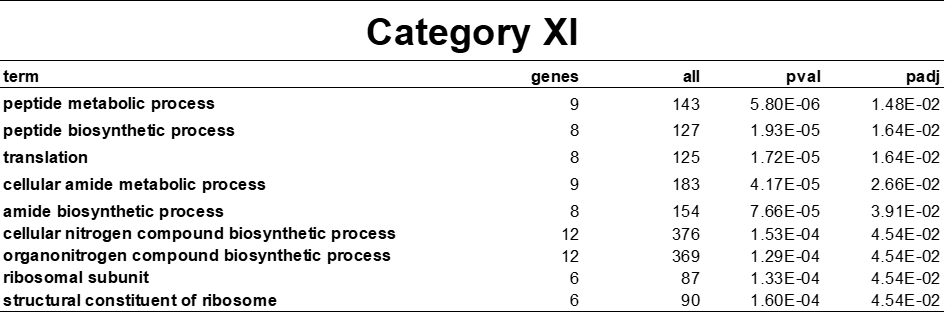


**Supplementary Table 4. Overrepresented GO terms in Category XI genes.** This table presents Gene Ontology (GO) terms that are significantly overrepresented among Category XI genes–those exhibiting a rapid decrease in expression during the laboratory natural selection experiment in the Chlamydomonas reinhardtii triploid lines. The terms are listed in ascending order of adjusted p-values to highlight the most statistically significant enrichments.

^
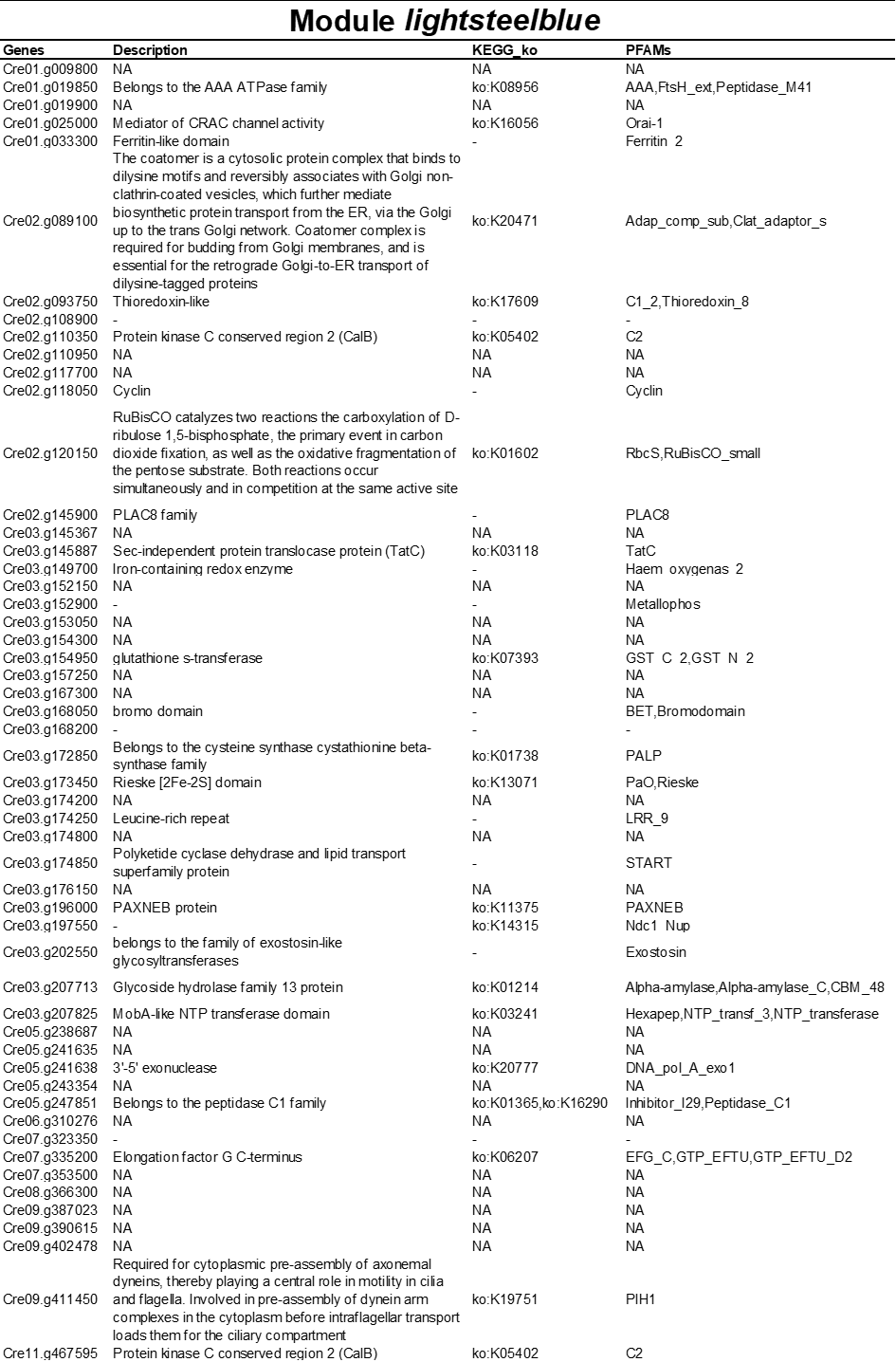
^

^
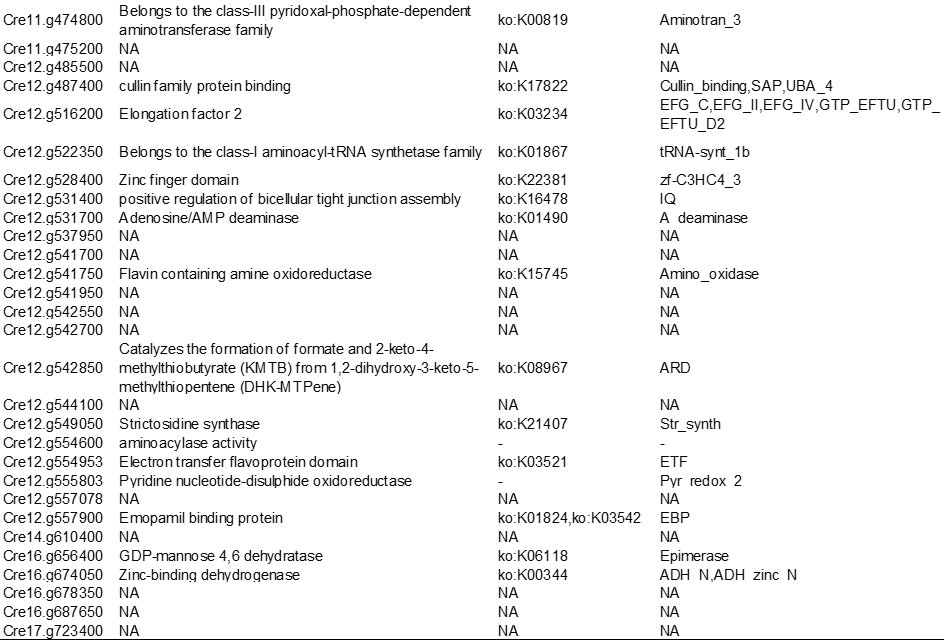
^

**Supplementary Table 5. Overview of genes in the lightsteelblue module.** This table lists the genes contained within the lightsteelblue module, along with their descriptions. Notably, the lightsteelblue module was the only one in the reference network that was not preserved across other test networks. Line plots (Fig. 6B) indicate that expression divergence between Line 1 and Line 4 emerged after generation 425, highlighting the unique evolutionary trajectory of this module.
